# Bioaccumulation of trace metals in octocorals depends on age and tissue compartmentalization

**DOI:** 10.1371/journal.pone.0196222

**Published:** 2018-04-23

**Authors:** Jiang-Shiou Hwang, Hans-Uwe Dahms, Ke Li Huang, Mu-Yeh Huang, Xue-Jun Liu, Jong Seong Khim, Chong Kim Wong

**Affiliations:** 1 Institute of Marine Biology, National Taiwan Ocean University, Keelung, Taiwan; 2 Dept. of Biomedical Science and Environmental Biology, Kaohsiung Medical University, Kaohsiung, Taiwan; 3 Department of Marine Biotechnology and Resources, National Sun Yat-sen University, Kaohsiung, Taiwan R.O.C; 4 Department of Marketing and Logistic Management, Yu Da College of Business, Miaoli, Taiwan; 5 Department of Biology, The Chinese University of Hong Kong, Shatin, N.T., Hong Kong, China P.R; 6 School of Earth and Environmental Sciences & Research Institute of Oceanography, Seoul National University, Seoul, Korea; Zhejiang University College of Life Sciences, CHINA

## Abstract

Trace metal dynamics have not been studied with respect to growth increments in octocorals. It is particularly unknown whether ontogenetic compartmentalization of trace metal accumulation is species-specific. We studied here for the first time the intracolonial distribution and concentrations of 18 trace metals in the octocorals *Subergorgia suberosa*, *Echinogorgia complexa* and *E*. *reticulata* that were retrieved from the northern coast of Taiwan. Levels of trace metals were considerably elevated in corals collected at these particular coral habitats as a result of diverse anthropogenic inputs. There was a significant difference in the concentration of metals among octocorals except for Sn. Both species of *Echinogorgia* contained significantly higher concentrations of Cu, Zn and Al than *Subergorgia suberosa*. We used for the first time exponential growth curves that describe an age-specific relationship of octocoral trace metal concentrations of Cu, Zn, Cd, Cr and Pb where the distance from the grip point was reflecting younger age as linear regressions. The larger colony (C7) had a lower accumulation rate constant than the smaller one (C6) for Cu, Zn, Cd, Cr and Pb, while other trace metals showed an opposite trend. The Cu concentration declined exponentially from the grip point, whereas the concentrations of Zn, Cd, Cr and Pb increased exponentially. In *S*. *suberosa* and *E*. *reticulata*, Zn occurred primarily in coenosarc tissues and Zn concentrations increased with distance from the grip point in both skeletal and coenosarc tissues. Metals which appeared at high concentrations (e.g. Ca, Zn and Fe) generally tended to accumulate in the outer coenosarc tissues, while metals with low concentrations (e.g. V) tended to accumulate in the soft tissues of the inner skeleton.

## Introduction

Coral reefs are fragile ecosystems, characterized by high biodiversity and high productivity [1]. In recent decades, coral reefs worldwide are impacted by global climate change and anthropogenic disturbances [2, 3, 4]. Three chemical pollution categories have generally attracted attention in marine systems: oil and oil derivatives [2, 5], heavy metals [6, 7], and synthetic organics such as pesticides and herbicides [8, 9]. The most important forms of anthropogenic disturbance to coral reefs include additional threats such as damages associated with fishing activities, euthrophication and pollution caused by sewage discharge, heat stress caused by effluents from power plants, and sedimentation from dredging and mud dumping (as reviewed by [10, 11, 12]).

The coral reefs around Taiwan are characterized by high species diversity [13]. According to some studies [14], this high species diversity is caused by unique oceanographic conditions that are partly due to the Kuroshio Current and seasonal monsoons [15, 16, 17, 18, 19]. Dai [13] recorded 230 species of anthozoans, representing 58 genera of scleractinian corals, 9 species of non-scleractinian reef building corals and 40 species of alcyonarian corals from Taiwan. During the last decades, intensive fishing activities and tourism have become major causes for the destruction of coral reef communities [14, 20]. Coastal areas around Taiwan are also intensively disturbed by urban development, industry, aquaculture and agriculture [12]. Pollutants from agricultural run-offs, urban areas, storm drains, and sewage effluents are discharged into rivers ending up eventually in the sea [21]. Along with other pollutants, metals accumulate in bottom sediments of rivers, estuaries, and mangrove ecosystems. When bottom sediments in coastal areas are dredged to open up channels for ships, or when mangrove forests are removed to provide areas for aquaculture, metals are resuspended and the health of coral reefs is threatened [3, 22]. Johannes [23] suggested that coral reefs in tropical regions have a narrow tolerance to fluctuations in physical and chemical parameters, and are highly susceptible to man-induced pollutants. A recent survey of Taiwan’s coastal areas revealed a general trend for the decline of coral species diversity and coral cover [24].

Octocorals are commonly found along rocky shores at water depths of 0−35 m. Octocorals are suspension feeders, prefering current velocities of 7−9 cm/s [25]. Many octocorals use calcified holdfast structures or long, rod-like internal support to attach to substrates. The outer layer or coenosarc consists of an epitheca and calcareous spicules, whereas the inner layer is composed of soft gorgonin which contains proteins and polysaccharides [26]. Since we expect a difference in the bioaccumulation of trace metals between both outer and inner layers, we sampled their tissues separately. Soft corals have a higher affinity to metals than hard corals and can be used to monitor the accumulation of heavy metals in their body, which in turn will effect their growth and reproduction [27, 28]. The diversity, health, and extent of coral cover and the geochemical make-up of their skeletons, can provide information on environmental changes in nearby coastal areas and oceans [29, 30, 31].

Trace metals are important pollutants in continental shelf regions and coastal zones [8, 32, 33, 34]. While some metals are essential to living organisms, several are toxic above certain threshold concentrations [35, 36, 37, 38]. High concentrations of trace metals have been reported generally for corals [39]. High concentrations of Cu were shown to affect corals letally [40]. It was shown that metals reduce the reproductive success of corals [41], inhibit the larval settlement of corals [42], and contribute to coral bleaching [43]. For most marine organisms, particularly for sessile suspension feeders like corals, suspended sediments are an important source of metal contamination [44].

In the past decades, industrial development has caused serious metal pollution, where metals accumulate in marine sediments [6, 7, 45, 46] and marine organisms [47, 48] in many coastal areas worldwide [12, 14, 49]. In Taiwan, investigators reported bio-accumulation of metals in plankton [50, 51], as well as in sediments and benthic organisms [11, 52, 53], and scleractinian corals [54]. Levels of heavy metals were considerably higher in seawater, sediments and corals collected from reef sites that were exposed to increased natural and anthropogenic contamination. Among corals, the ontogenetic history of metal bio-accumulation can be monitored in the annual growth zones of some scleractinian coral skeletons [19, 55, 56, 57]. Although many soft corals can grow in highly turbid and metal contaminated waters in Taiwan [58], the bio-accumulation of metals in soft corals has never been investigated here before.

The general aim of this study was to evaluate whether octocorals, especially *Subergorgia suberosa*, *Echinogorgia complexa*, and *E*. *reticulata*, are suitable for the monitoring of trace element pollution. The particular objectives of the present study were: 1). to determine the concentrations of 18 trace metals in 3 octocorals at the northern coast of Taiwan; 2). assuming that bioaccumulation occurs, to investigate the relationship between metal concentration and growth of octocorals by measuring metal concentrations at different distances from the oldest colony part, the grip point; 3). to analyze the compartmentalization of metals in the outer coenosarc tissues and the soft inner layer.

## Materials and methods

### Study sites

**Fig 1:**
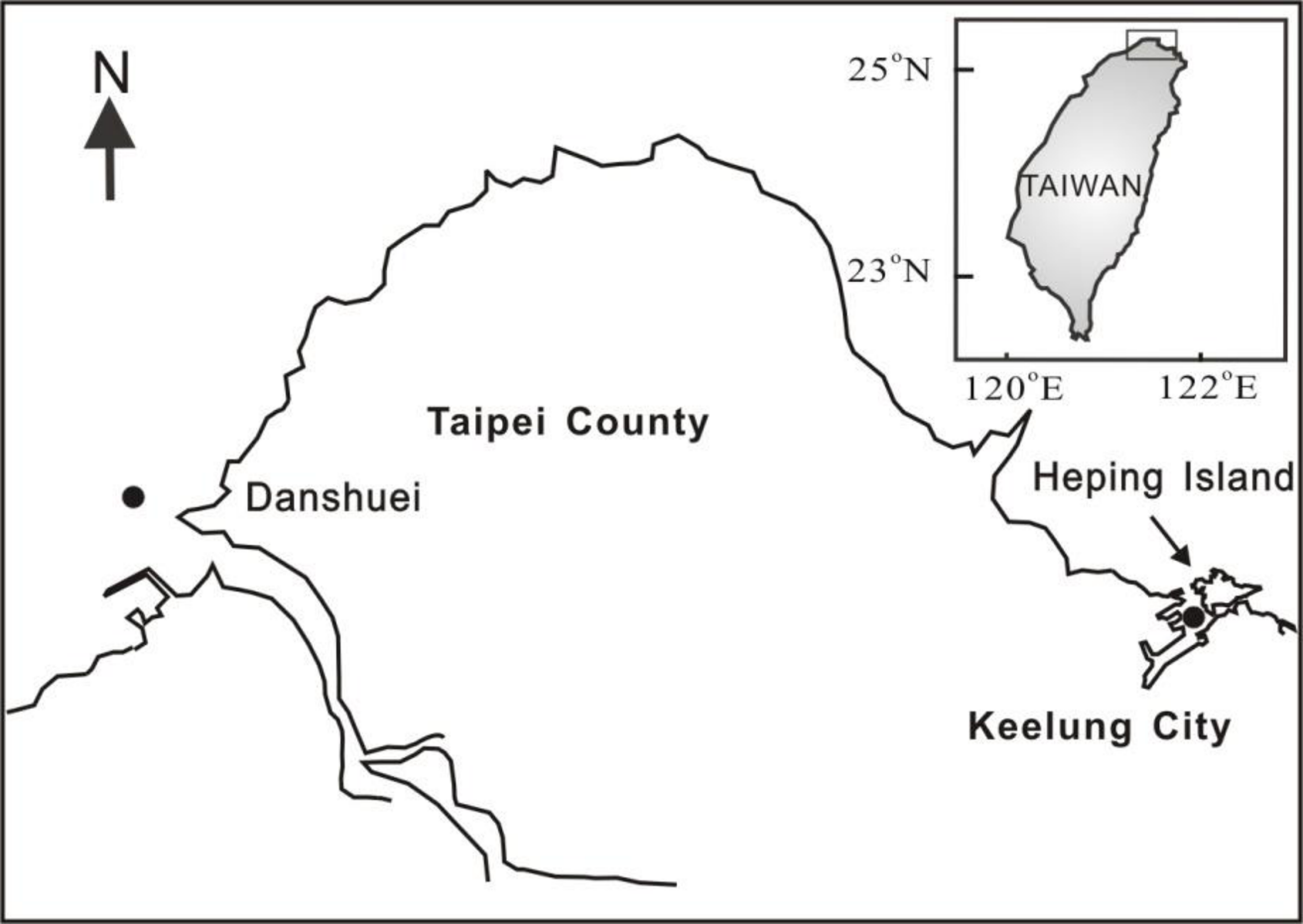
Sampling locations at Danshui estuary and Heping Island, northern Taiwan.

Corals were collected from the Danshuei estuary and from Heping Island in northern Taiwan (**Fig 1**). The low-density soft coral community in the Danshuei estuary is dominated by several species of octocorals. The coastal areas around Heping Island were covered scarcely and by gorgonians only. Otherwise, there were only rare patches of scleractinian corals in northern Taiwan.

### Samples

Since the samples for this study were not taken in a national park or other protected area of land or sea, nor on private land, we did not have to ask any regulatory body or authority concerned with protection of wildlife, etc. for permission. We state clearly that no specific permissions were required for our activities at the locations above since they were publicly accessible parts of government owned outer harbor areas. We further confirm that neither our field inventories nor the samples taken for laboratory experiments involved any endangered or protected species.

*Echinogorgia reticulata* was collected in the Danshuei estuary in May 1999. *Subergorgia suberosa* and *E*. *complexa* were collected from Heping Island in June 1999. Samples collected at the two sites by SCUBA divers included 7 colonies of *S*. *suberosa* (species A), 7 colonies of *E*. *complexa* (species B) and 9 colonies of *E*. *reticulata* (species C). Water depths were ~20 m in the Danshuei estuary and ~12 m at Heping Island. Corals were picked up from the grip point at the sediment / water interface. Collected corals were generally treated according to procedures described by [56]. Sediment on the surface of corals was removed by a soft brush. The specimens were then rinsed with distilled water and returned to the laboratory where the remaining mud and clay was removed by sonication. Cleaned specimens were blotted and dried before the length, width and weight of each colony were measured. Tissue samples for measurement of metal concentrations were divided into 4 categories based on the distance from the grip point: trunk (a1, a2, …), first-order branch (b1, b2, …), second-order branch (c1, c2, …), and third-order branch (d1, d2, …).

### Analytical methods

Coral skeletons were thoroughly cleaned to remove surface sediments and symbiotic zooxanthellae before cutting them into pieces according to the above procedure. Then, coenosarc tissues were laterally cut off from the inner layer and both samples were treated separately. All samples were subjected to acid digestion (HNO_3_, analytical grade) [58]. Finally, the concentrations of 18 trace metals (Ca, Al, Zn, Cu, Fe, Mn, Ni, Se, As, Ba, Ag, Au, Cd, Co, Cr, Pb, Sn and V) were measured using the digested samples. Considering the low ratio of Mg to Ca (ca. 0.25% [59]), an EDTA-titration method [60] was used to measure the concentration of Ca. Concentrations of Cu, Zn, Fe, and Mn were measured with Flame Atomic Absorption Spectrometer (FAAS) (Hitachi Z-6100, Tokyo, Japan). Concentrations of Cd, Cr and Pb were measured with Graphite Furnace Atomic Absorption Spectrometer (GFAAS) (model: Perkin-Elmer 5100, Greenville, SC, USA) equipped with Zeeman background corrections. Concentrations of the other 10 metals were measured with ICP-MS (Perkin-Elmer ELAN 5000, Greenville, SC, USA).

### Quality control and statistics

Metal standards and standard reference materials were used to monitor the accuracy and precision of metal analyses. Metal recovery ranged from 93 to 118% and variations among replicate analyses were <10%. Procedural blanks were lower than the detection limits for Cd, Zn, Hg and Mn. Blanks for Al were <4% of the measured concentrations. ANOVA and Duncan’s Multiple Range Test (DMRT) were used to test for differences in the concentration of metals among 3 octocorals. The degree of the association between metal accumulation and coral growth was determined using a semi-natural logarithmic linear regression analysis (exponential growth curve). Linear correlations were calculated for metal accumulation between skeleton and coenosarc tissues. All statistical analyses were conducted using SigmaStat v3.1 (Systat Software Inc., CA, USA).

## Results

### Comparison of metal concentrations among 3 octocorals

**Table 1** lists the mean concentrations of 18 metals and the concentrations of these metals in proportion to calcium in the octocorals, *S*. *suberosa* (A1 to A5), *E*. *complexa* (B1 to B5), and *E*. *reticulata* (C1 to C5) (**Table 1**). Except for Sn, the concentrations of all metals varied among the 3 species of octocorals (p < 0.01 for Cu, Zn, Fe, Mn, Ag, As, Ba, Cd, Co, Ni, Pb, Se and V; p < 0.05 for Ca, Al, Au and Cr) and their tissues. *E*. *reticulata* retrieved from estuarine waters and *E*. *complexa* from coastal marine waters showed quite similar metal concentrations. The concentrations of Al, Zn and Cu were much higher in the two species belonging to *Echinogorgia* compared to *S*. *suberosa*.

**Table 1:** Concentrations of 18 metals (mean ± SE, n = 5) in proportion to calcium concentrations in *S*. *suberosa*, *E*. *complexa* and *E*. *reticulata* of combined, soft inner layer and coenosark.

**Metals**	***S*. *suberosa***		***E*. *complexavc***		***E*. *reticulata***	
	Concentration	Metal conc./ Ca conc.	Concentration	Metal conc./ Ca conc.	Concentration	Metal conc./ Ca conc.
	(μg/g DW)	(10^−6^)	(μg/g DW)	(10^−6^)	(μg/g DW)	(10^−6^)
Ca^†^	306.1 ± 9.7	-	267.2 ± 5.4	-	269 ± 10	-
Al	49.9 ± 1.3	163.02	144 ± 20	538.92	117 ± 26	434.94
Zn	18.4 ± 1.5	60.11	249 ± 43	931.89	201 ± 18	747.21
Cu	2.51 ± 0.37	8.20	81.7 ± 8.9	305.76	83 ± 13	308.55
Fe	7.52 ± 0.51	24.57	22.5 ± 1.9	84.21	27.0 ± 5.5	100.37
Mn	3.98 ± 0.13	13.00	13.3 ± 1.41	49.78	24.1 ± 3.11	89.59
Ni	6.30 ± 0.27	20.58	5.47 ± 0.15	20.47	7.58 ± 0.29	28.18
Se	7.60 ± 1.00	24.83	8.05 ± 0.33	30.13	13.6 ± 2.1	50.56
As	6.27 ± 0.66	20.48	7.59 ± 0.87	28.41	4.19 ± 0.85	15.58
Ba	4.28 ± 0.14	13.98	4.00 ± 0.18	14.97	7.56 ± 0.85	28.10
Ag	0.04 ± 0.02	0.13	0.16 ± 0.03	0.60	0.06 ± 0.01	0.22
Au	0.05 ± 0.01	0.16	0.13 ± 0.03	0.49	0.02 ± 0.01	0.07
Cd	0.44 ± 0.03	1.44	1.89 ± 0.14	7.07	0.93 ± 0.11	3.46
Co	0.33 ± 0.01	1.08	0.46 ± 0.02	1.72	0.56 ± 0.04	2.08
Cr	0.30 ± 0.02	0.98	0.62 ± 0.10	2.32	0.58 ± 0.11	2.16
Pb	0.60 ± 0.05	1.96	1.30 ± 0.11	4.87	1.60 ± 0.18	5.95
Sn	0.40 ± 0.12	1.31	0.20 ± 0.02	0.75	0.13 ± 0.02	0.48
V	0.38 ± 0.02	1.24	2.15 ± 0.27	8.05	1.34 ± 0.19	4.98

^†^ in mg/g DW.

Based on the results of DMRT, metals in each gorgonian were grouped according to their concentrations. In *S*. *suberosa*, the metals could be separated into 4 groups with Ca (306.1 mg/g DW) showing the highest concentration, followed by Al (49.9 μg/g DW), Zn (18.4 μg/g DW), and all other metals (0.03–7.6 μg/g DW). In *E*. *complexa*, 5 groups were identified, with Ca (267.2 mg/g DW) occurring at the highest concentration, followed by Zn (248 μg/g DW), Al (1441.1 μg/g DW), Cu (81.7 μg/g DW) and then by all other metals (0.13–22.5 μg/g DW). Metals in *E*. *reticulata* were separated into 5 groups with Ca (270 mg/g DW) having the highest concentration, followed by Zn (201 μg/g DW), Al (117 μg/g DW), Cu (82.9 μg/g DW), and all other metals studied here (0.02–27.0 μg/g DW).

#### Comparison of metal partitioning in three species of octocorals

Partitioning of metals in octocorals was studied by comparing metal concentrations between the outer coenosarc tissues and the soft inner layer (**Table 2**). In all 3 octocorals, the concentrations of Cu, Se and V were higher in the outer coenosarc tissues than in the soft inner layer tissues, while the concentrations of Ca, Zn, Fe, Mn, Cr, Ba and Ni were higher in the outer coenosarc than in the inner layer. No clear pattern was observed in the distribution of the other metals (Al, Ag, As, Au, Cd, Co, Pb and Sn), but the coenosarc to inner layer ratios in metal concentrations tended to be >1.0 for most metals. In general, metals which appeared at high concentrations (e.g. Ca, Zn and Fe) tended to accumulate in the outer coenosarc tissues, while metals which appeared at low concentrations (e.g. V) tended to accumulate in the inner layer tissues.

**Table 2:** Concentrations (mean values in μg/g DW, n = 5) of 18 metals in soft inner layer and coenosarc of *S*. *suberosa*, *E*. *complexa* and *E*. *reticulata*.

**Metals**	*S*. *suberosa*			*E*. *complexa*			*E*. *reticulata*		
	Soft inner layer	Coenosarc	Coenosarc/ soft inner layer	Soft inner layer	Coenosarc	Coenosarc/ soft inner layer	Soft inner layer	Coenosarc	Coenosarc/ soft inner layer
Cu	2.31	1.57	0.68	73.3	4.56	0.06	98.4	5.23	0.05
Ca^†^	107	255	2.38	128	211	1.65	81.5	242	2.97
Zn	8.74	25.5	2.91	30.6	168	5.50	53.9	189	3.51
Fe	1.47	8.23	5.59	4.15	23.7	5.72	16.1	37.7	2.35
Mn	2.93	3.31	1.13	4.46	11.6	2.61	10.7	13.0	1.21
Al	1.01	42.6	42.2	88.7	132	1.49	56.8	39.1	0.69
Ag	ND^‡^	0.02	-	0.13	0.24	1.84	0.07	0.30	4.33
As	2.03	7.70	3.79	3.35	6.73	2.01	3.85	2.56	0.66
Au	ND	0.04	-	0.21	0.04	0.18	0.63	0.03	0.04
Ba	2.06	2.77	1.34	ND	4.82	-	0.50	10.1	20.4
Cd	0.35	0.54	1.52	0.68	1.66	2.47	1.56	1.01	0.65
Co	0.18	0.29	1.65	0.98	0.34	0.35	0.68	0.48	0.71
Cr	0.27	0.31	1.15	0.15	0.45	3.00	0.28	0.73	2.63
Ni	3.15	3.69	1.17	2.15	4.00	1.86	3.09	6.47	2.10
Pb	0.31	0.38	1.24	0.74	0.50	0.67	0.62	2.91	4.70
Se	10.2	1.39	0.14	8.70	2.27	0.26	11.18	6.30	0.56
Sn	0.34	1.01	2.95	0.28	0.15	0.55	0.24	0.18	0.76
V	0.36	0.35	0.98	2.30	0.44	0.19	1.75	0.69	0.39

^†^ in mg/g DW

^‡^ not detected.

#### Relationship between coral growth and metal accumulation

**Fig 2:**
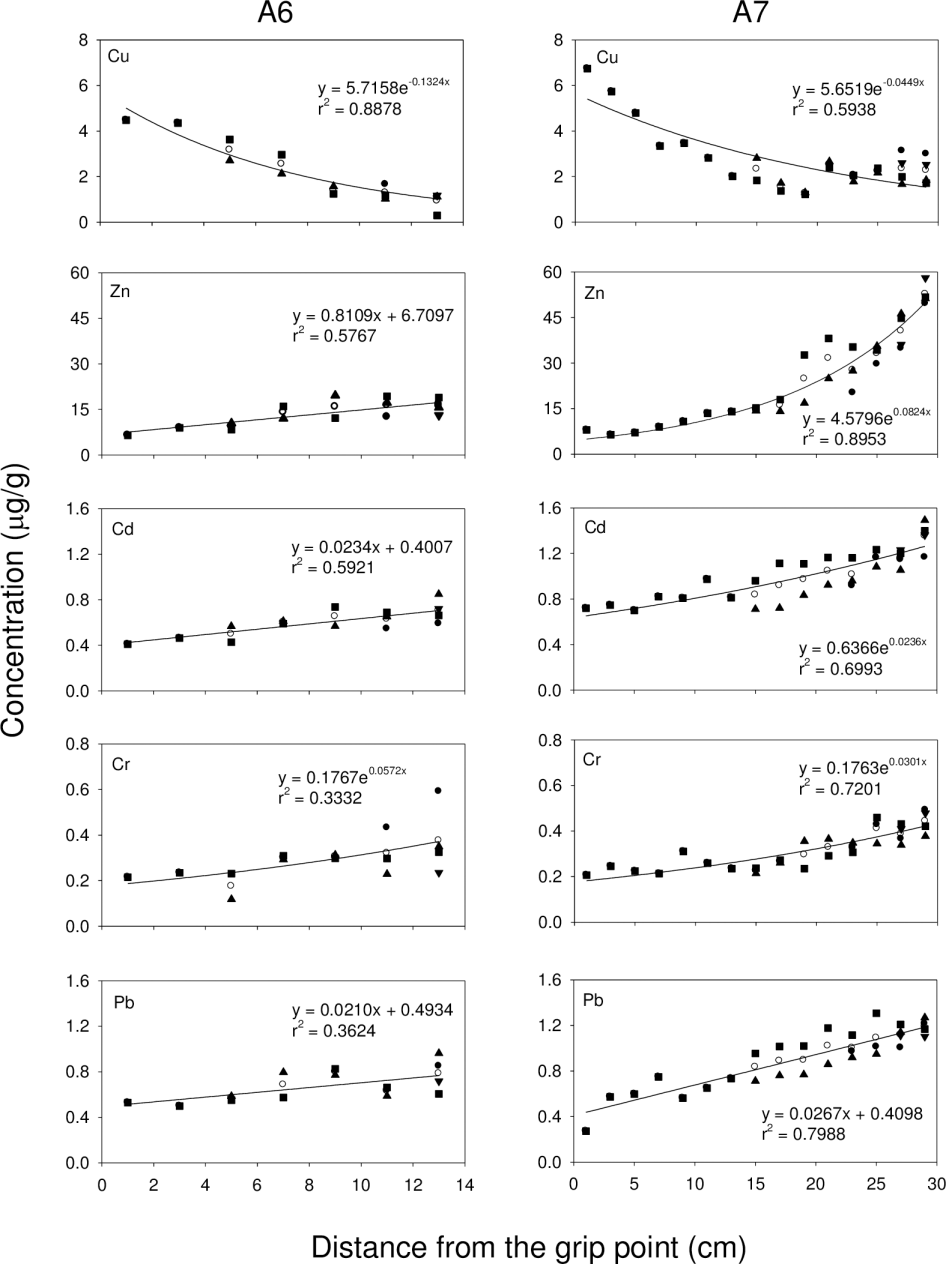
Relationship between the concentrations of Cu, Zn, Cd, Cr and Pb and the distances from the grip point in two colonies (A6 and A7) of *S*. *suberosa* (●trunk, ■1^st^-order branch, ▲2^nd^-order branch, ▼3^rd^-order branch and ○ mean).

**Fig 3:**
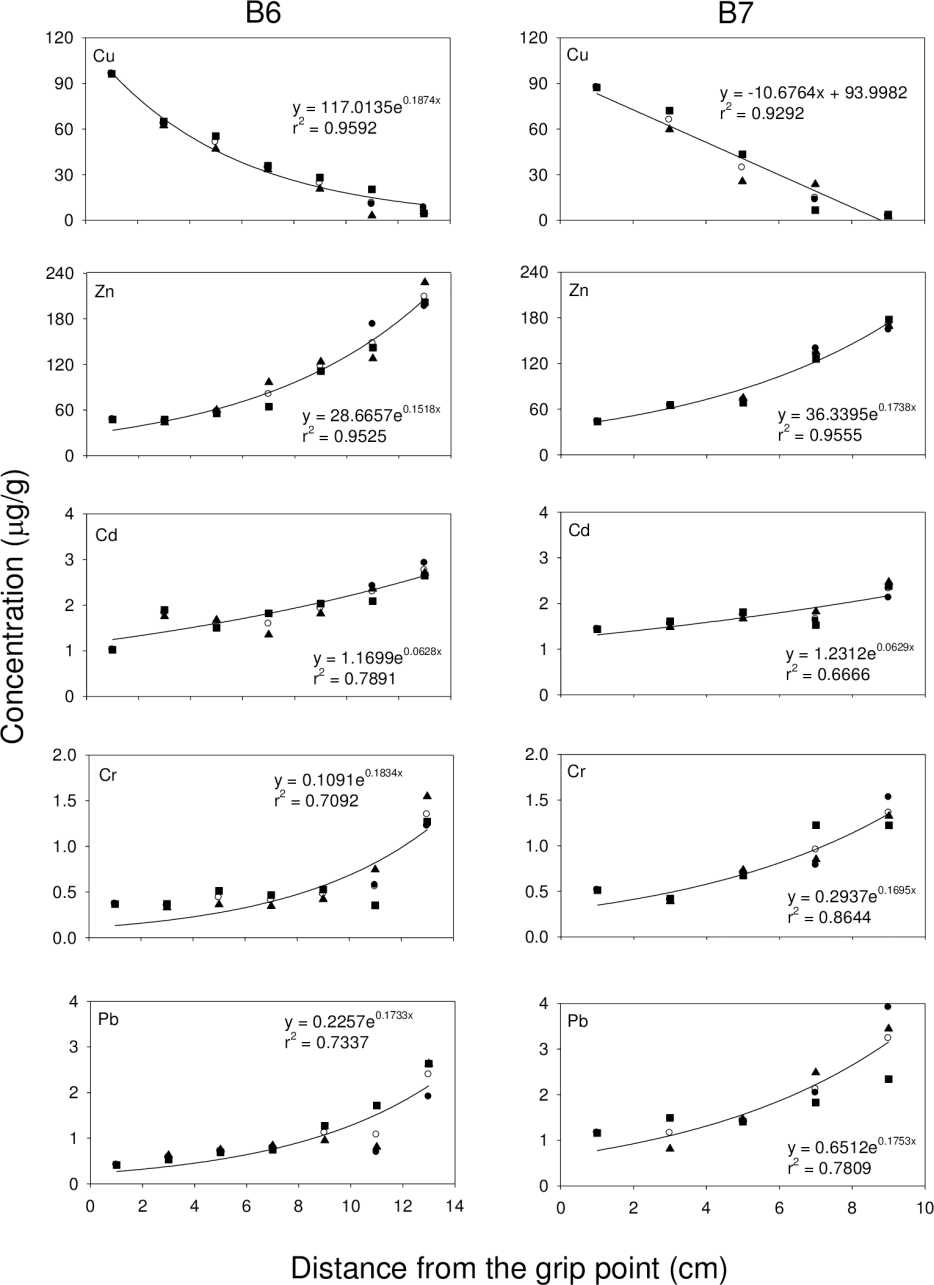
Relationship between the concentrations of Cu, Zn, Cd, Cr and Pb and the distances from the grip point in two colonies (B6 and B7) of *E*. *complexa* (●trunk, ■ 1^st^-order branch, ▲ 2^nd^-order branch and ○ mean).

**Fig 4:**
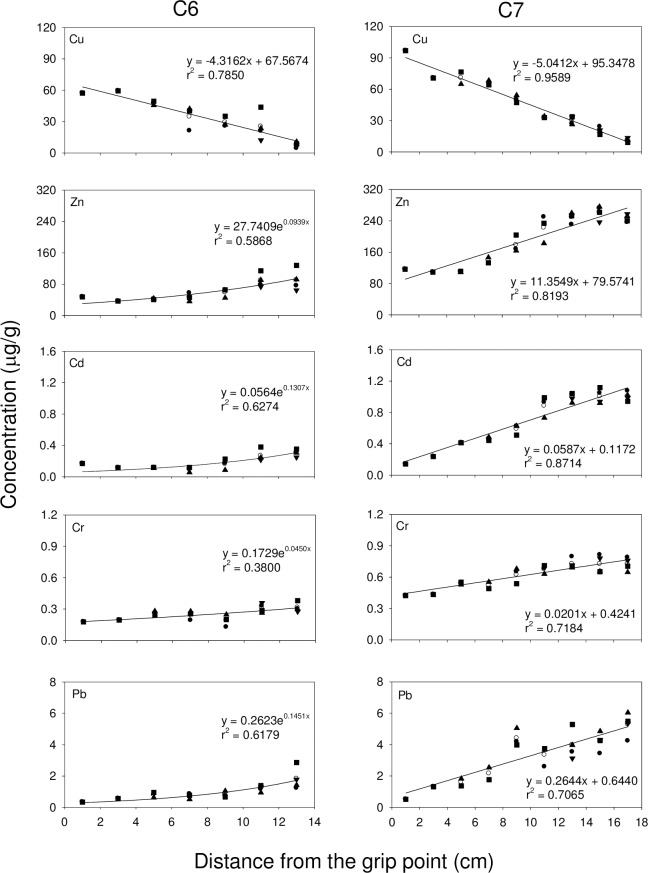
Relationship between the concentrations of Cu, Zn, Cd, Cr and Pb and the distances from the grip point in two colonies (C6 and C7) of *E*. *reticulata* (● trunk, ■ 1^st^-order branch, ▲ 2^nd^-order branch, ▼ 3^rd^-order branch and ○ mean).

Exponential growth curves are commonly used to study the relationship between the concentrations of metals and the distance from the grip point in different branches. In all 3 octocoral species, the concentration of Zn, Cd, Cr and Pb increased with distance from the grip point, while the concentration of Cu decreased with distance from the grip point (**Figs 2, 3,** and **4**). For *S*. *suberosa*, the larger colony (A7) exhibited a lower rate constant in the accumulation of metals than the smaller colony (A6), except for Zn. The same trend was also applicable to *E*. *complexa* and *E*. *reticulata*. However, the larger colony (B6) of *E*. *complexa* had a higher accumulation rate constant than the smaller one (B7) for Cd (**Fig 3**). In *E*. *reticulata*, a modified exponential growth model was employed to describe the relationship between the distance from the grip point and the accumulation of the 5 metals mentioned above (**Fig 4**). It is worth noting that without exception, the larger colony (C7) showed higher concentrations than the smaller one (C6) of Cu, Zn, Cd, Cr and Pb.

#### Relationship between growth and Zn partitioning in three coral species

**Fig 5:**
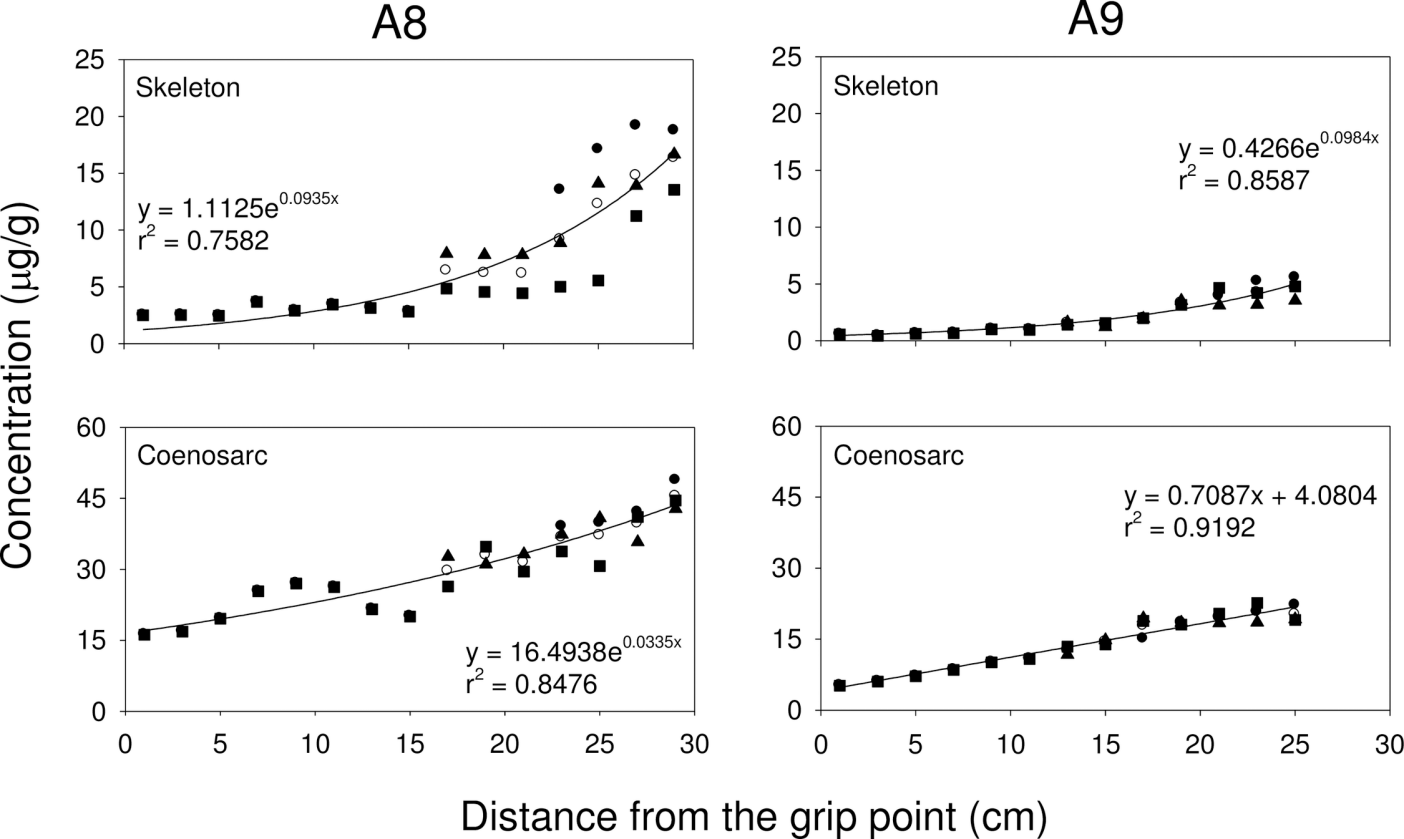
Zn accumulation in skeleton and coenosarc tissues in *S*. *suberosa* associated with distance from the grip point (● trunk, ■ 1^st^-order branch, ▲ 2^nd^-order branch and ○ mean).

**Fig 6:**
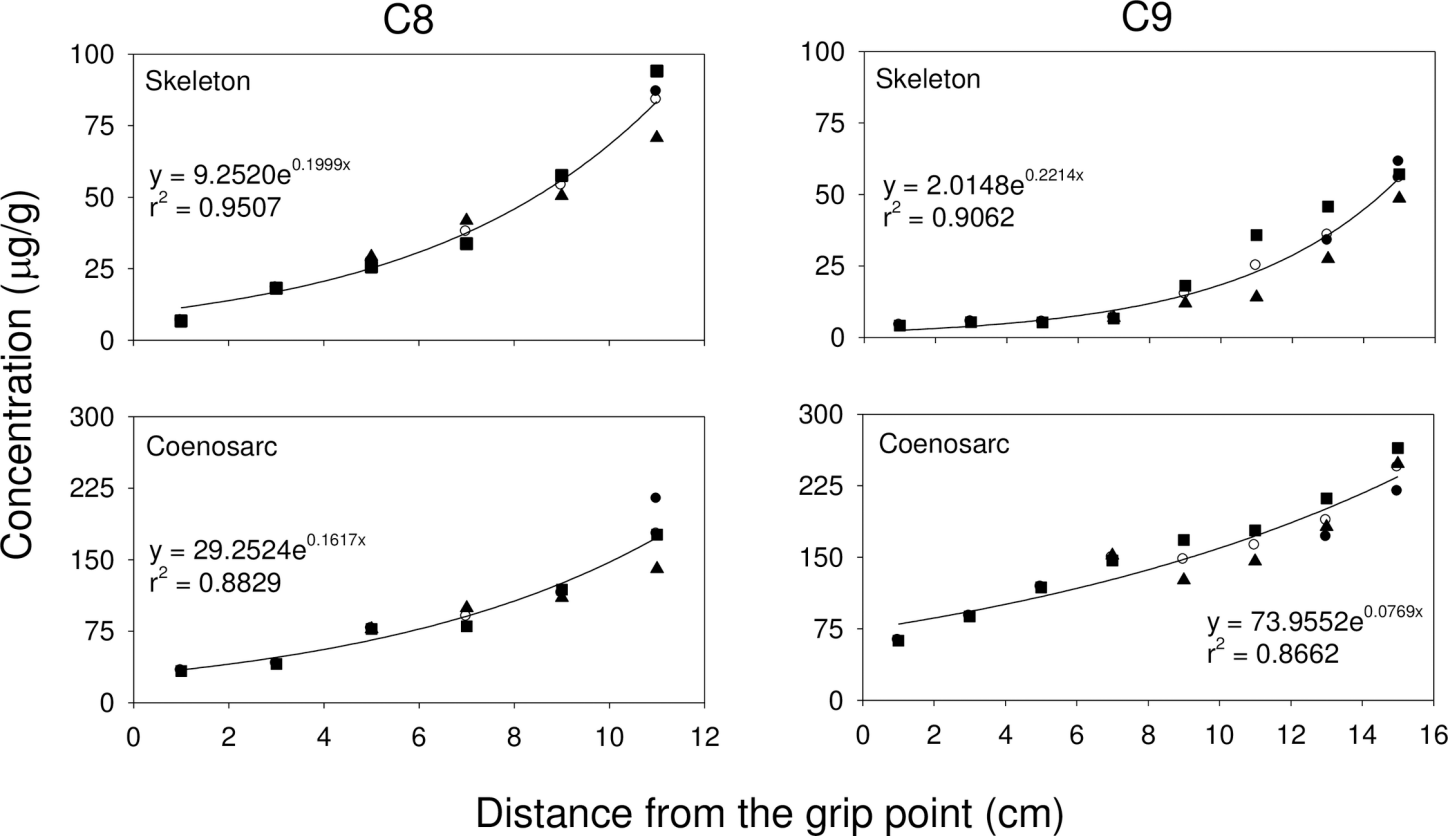
Zn accumulation in skeleton and coenosarc tissues in *E*. *reticulata* associated with distance from the grip point (● trunk, ■ 1^st^-order branch, ▲ 2^nd^-order branch and ○ mean).

Zn was the second most abundant metal in the octocorals studied here. The relationships between distance from the grip point and Zn concentrations were examined in skeletal and coenosarc tissues in all 3 species of octocorals. For *S*. *suberosa*, Zn concentrations increased with distance from the grip point in both types of tissues (**Fig 5**). A similar pattern was found in *E*. *reticulata* (**Fig 6**). In both, *S*. *suberosa* and *E*. *reticulata*, Zn concentrations were higher in coenosarc tissues than in the inner soft parts. A comparison of Zn concentrations in the coenosarc and inner soft layer suggests that Zn from the ambient environment was incorporated by *E*. *reticulata* at higher concentrations than by *S*. *suberosa*.

**Fig 7:**
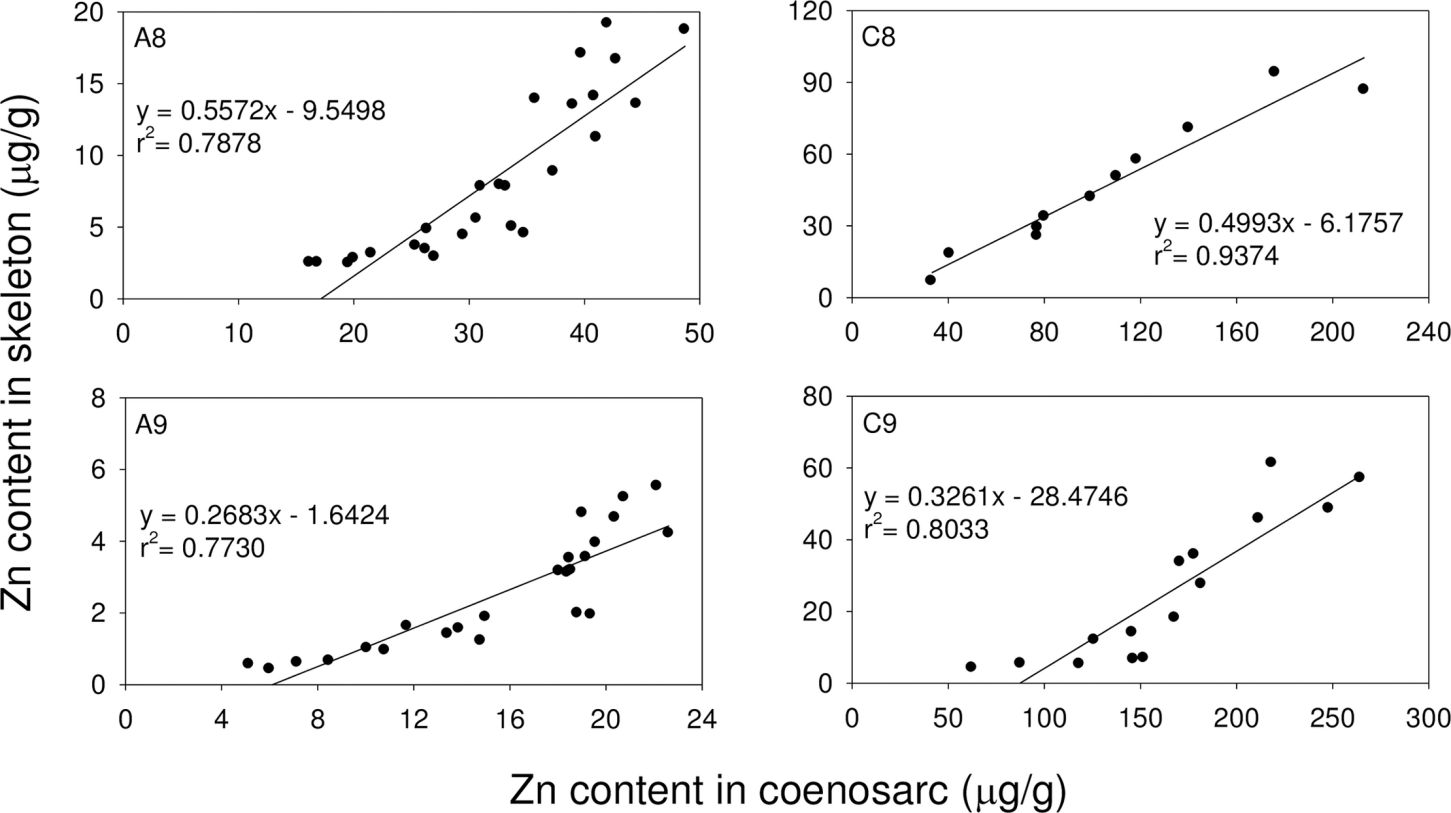
Linear regressions between Zn content in skeleton and coenosarc tissues in *S*. *suberosa* and *E*. *reticulata*.

A linear regression model was applied to study the relationship between Zn concentrations in skeletal and coenosarc tissues of *S*. *suberosa* and *E*. *reticulata* (**Fig 7**). In both species, Zn concentrations in the two types of tissues correlated strongly. In addition, Zn concentrations were higher in coenosarc tissues than in the soft inner tissues.

## Discussion

The concentrations of Al, Zn and Cu were generally higher than those of other metals in the octocorals studied here. This pattern probably reflected the concentrations of metals in the water column and the sediment and a differential accumulation pattern of different metals by different coral species [35].

Cu and Zn concentrations in corals recorded in the present study were higher than those recorded in corals from the Great Barrier Reef [61, 62], Venezuela [28], and along the Egyptian coast of the Red Sea [53]. Denton and Jones [61] reported that variability of Cu concentrations in both soft and hard corals were smaller than those of Zn and Cd and concluded that the accumulation and depuration processes for Cu might be quite different from those for Zn and Cd. Our results (**Figs 2, 3** and **4**) showed that the concentration of Cu decreased with distance from the grip point, while the concentrations of Zn and three other non-essential metals (Cd, Cr and Pb) increased.

Such concentration differences might be related to differences in the relative contribution of aqueous and sedimentary phases to the overall bioaccumulation of metals. Higher concentrations of Zn, Cd, Cr and Pb at the lower and inner reef areas are explained by the likely higher ambient metal contents in these areas that were polluted by river run-offs [46]. In contrast, higher concentrations of Cu near the grip point could primarily be the result of high Cu levels from sediments [12].

According to [63], is Zn particularly taken up by growing aragonite crystals. So, Zn was suggested as a pollution indicator in the Hija River (Japan). A related study revealed that Zn concentrations in coral skeletons could be 11 folds higher than those in ambient seawater [58].

The mechanism of Cu accumulation in organisms remains largely unknown. Cu compounds are rather complex due to their ability to bind with various ligands [64]. However, Cu accumulation might be associated with the bioaccumulation of other metals, demonstrating synergistic or antagonistic effects. However, our results are consistent with the finding that Cu decreases in demersal or sessile organisms [65]. The relationship between distance from the grip point and metal concentrations suggests that the concentrations of Cu, Zn, Al, Fe and Mn increases with age of the different growth zones from the grip point due to bioaccumulation. Zn and Cu are both essential metals. Zn is a key component of many enzymes including carbonic anhydrases [66], and Cu is a functional part of the respiratory protein haemocyanin [67]. A certain low concentration of these two metals is required to maintain essential metabolic activity. It is thus, possible to make theoretical estimates of essential concentrations, and any additional accumulation of these metals would be followed by excretion, storage and/or detoxification. In contrast, no minimum concentration occurs for non-essential metals such as Cd, Cr and Pb. These metals are excreted from the body as part of coral detoxification mechanisms [35]. This could explain our finding that Zn tends to be accumulated by the specific-tissues, in different concentrations in octocorals.

When Zn is taken up by the organism and incorporated at cellular or subcellular levels, it would eventually bind to sulphur-containing proteins (such as metallothionin and transferrin-like proteins). It subsequently forms some granules inside the organism [66]. Based on this observation, Zn and possibly other metals are prone to be taken up in tissues with a high sulphur content. In our study Zn shows higher concentrations in coenosarc tissues than in the inner soft layers of *S*. *suberosa* and *E*. *reticulata* (**Figs 5** and **6**).

The present study surveys the concentrations of 18 metals in 3 species of common octocorals in northern Taiwan. Metals are important marine pollutants in northern Taiwan. In recent decades, the coastal areas around Heping Island at the northern Taiwan coast are disturbed by tourism and industrial and agricultural activities. Increased anthropogenic activities led to pollution of the coastal environment. Major sources of metals include untreated and semi-treated sewage effluents, anti-fouling paints, and discharge from agricultural and industrial developments. According to a routine survey conducted during 1995–1998, water quality around Heping Island was categorized as Class II polluted waters [47]. EPA–Taiwan [48] provided permissible levels in **Table 2** of their “Surface Water Classification and Water Quality Standards”. The most toxic trace metals should not be above a microgram thereshold (5ug/L for cadmium, 1 ug/L for mercury–both were not studied in here). However, the permissible levels of other trace metals were in the milligram range (o.1mg/L for nickel and 0.5 mg/L for zinc).

Many marine organisms accumulate metals at concentrations 1 to 4 orders of magnitude higher than environmental background concentrations [30]. Accumulation of metals can lead to extremely high tissue concentrations and toxic effects in a variety of marine organisms [67], including corals [68]. There are several studies on metal pollution in bivalves and fish in Taiwan, but the mechanisms of toxic action on corals are relatively unknown as yet [63].

The present study showed that the metal concentrations in 3 octocorals from northern Taiwan were relatively high compared to the surrounding sediments and water column reported in the literature [25]. Such were likely caused by long-term metal input from urbanized areas through river run-offs to coastal areas like the Danshui estuary or Heping Island (Keelung, Taiwan). These show a strong association with among others: metal availability in aqueous and sedimentary phases, chemical metal properties and kinetics, and coral reef growth. The partitioning of metals within corals occurred at higher concentrations in the coenosarc than in the inner soft tissues. Since the documented metal concentrations within corals reflect their interaction with the ambient environment, they can be used to monitor environmental changes with time. The present results are thus helpful for policy makers to establish input limits and enforce effluent criteria for the northern coast of Taiwan, or at other places, in order to protect the remaining coral reefs in this region. There are pressing needs for detailed information about the relative quantitative importance of contributions from the aqueous and sedimentary phases. The same holds for a delineation or compartmentalization of metals (particularly toxic heavy metals) at cellular or subcellular levels, and the possible interaction of different metals during accumulation and depuration processes. These efforts should be made in order to obtain a better understanding of coral health.
